# A compact MIMO antenna with high gain and dual circular polarization using a T divider for WLAN applications

**DOI:** 10.1038/s41598-025-05820-5

**Published:** 2025-07-01

**Authors:** Dat Tran-Huy, Nguyen Tran-Viet-Duc, Hung Tran, Niamat Hussain

**Affiliations:** 1 Faculty of Electrical and Electronic Engineering, PHENIKAA University, Hanoi, 12116 Vietnam; 2 Department of Convergence Engineering for Intelligent Drone, Sejong University, Seoul, 13391 Republic of Korea; 3 James Watt School of Engineering, University of Glasgow, Glasgow, G12 8QQ Scotland, UK

**Keywords:** Electrical and electronic engineering, Software

## Abstract

This paper presents a circularly polarized (CP) multiple-input multiple-output (MIMO) antenna with compact size and high-gain features for wireless local area network (WLAN) applications. The proposed approach employs two compact dual-CP antennas and T-junction power dividers to design 2-port MIMO antenna. The use of T-junction divider can excite both radiators simultaneously, resulting in high gain operation. For validation, an antenna prototype with overall dimensions of 1.06 $\lambda$ $\times$ 0.65 $\lambda$ $\times$ 0.03 $\lambda$ at 2.45 GHz is fabricated and measured. The measured operating bandwidth is from 2.43 to 2.485 GHz, in which the matching is less than $-10$ dB, the isolation is higher than 10 dB, and the axial ration is smaller than 3 dB. Additionally, the antenna also performs high gain radiation of about 8.0 dBi and good MIMO diversity performance in terms of envelop correlation coefficient, diversity gain, and so on. In comparison with the related works, the proposed antenna is beneficial in terms of CP radiation and overall dimensions with less number of required radiating elements, while achieving comparable performance.

## Introduction

Multiple-input-multiple-output (MIMO) antenna is one of the most important part in any modern wireless communication systems as it can provide high data-rate without the need for additional spectrum^1^. The MIMO antennas should be compact for an ease of integraton and high gain for long-distance communication. Besides, circularly polarized (CP) antenna is also prefer to solve the multipath interference problem, rather than linearly polarized (LP) antenna.

Various CP MIMO antennas have been reported in the open literature^2–10^. In such designs, the MIMO configurations are generally a combination of multiple single-polarized radiators. These MIMO elements are decoupled by using bandstop filters formed by defected ground structure or metamaterial. However, this design approach has a drawback of low gain radiation, which is commonly less than 7 dBi. The reason behind is that there only one radiating element for one port MIMO.

For gain improvement, the common approach is the use of multiple radiating elements. It means that for one port MIMO, multiple radiating elements are excited simultaneously, rather that only one element as the conventional designs^2–10^. In^11–15^, one MIMO port consists of T-junction power dividers connected to either two or four radiating patches. These antennas are able to realize LP wave with high gain from 8.0 to 14 dBi, depending on the number of radiating elements. Similar approach to design high gain MIMO antenna can be found in^16–19^, but with CP radiation. To conclude, despite having high gain performance, these antennas use the same method of combining T-divider and single-polarized radiator. It means that for 2-port MIMO system, at least four radiators are required. Definitely, it is not suitable for a large-scale MIMO array with compact size due to the requirement for a huge number of radiators. Another approach to improve the gain of the MIMO system without using power divider employs metasurface or frequency selective surface (FSS)^20–23^. However, this approach has a critical drawback of extremely high profile as the FSS layer has to be positioned above the radiating elements.

This paper proposed a method to design high gain CP MIMO antenna with small number of radiators to solve the current limitations of^11–19^ and low-profile configuration to overcome the drawback of^20–23^. Here, instead of using single-polarized radiators and T-dividers combination, dual-polarized radiators are chosen. By doing so, the number of required radiators can be reduced by half. For example, the method utilized in^11–19^ requires four radiators for 2-port MIMO antenna. Meanwhile, using the proposed method only needs two radiators. It is noted that dual-polarized patch has been used to design compact MIMO system, but the antenna in^24,25^ works with LP radiation.

## Compact dual-CP antenna

**Fig. 1:**
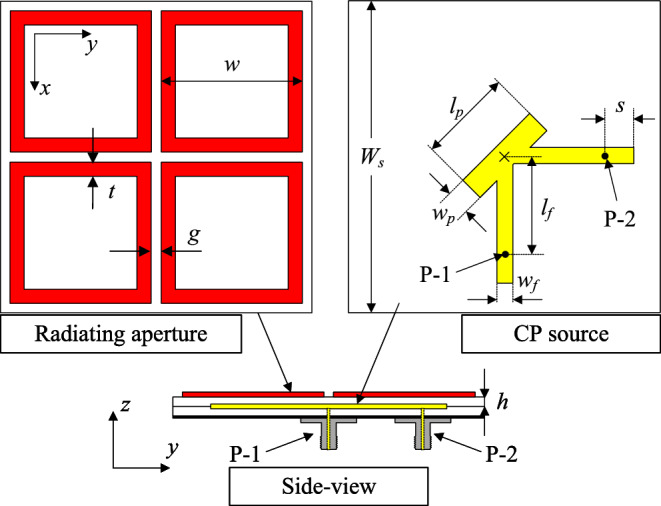
Geometry of the dual-CP antenna.

It is noted that this work is further development from our previous work reported in^25^. According to^25^, a working principle of a dual-CP antenna has been thoroughly investigated and thus, it is not shown here for brevity. Based on this work, a dual-CP antenna with capability of realizing right-hand CP (RHCP) and left-hand CP (LHCP) radiations is presented in Fig. 1. The antenna consists of a CP source, which is an asymmetric structure with capability of producing orthogonal fileds, and a radiating aperture. The operating frequency of this aperture is defined using Characteristic Mode Analysis, which has been discussed in^25^. These parts are printed on two different Taconic RF-35 substrates with a dielectric constant of 3.5. To achieve compact size, the radiating aperture consists of four square-ring-shaped patch, rather than square-shaped patch. The reason behind is that the the use of ring-shaped patch can extend the electrical length of current flowing on it. Two coaxial cables are utilized to excited the CP source at different feeding positions, designated as P-1 and P-2. When P-1 is excited, the antenna produce LHCP waves and vice versa, RHCP is the dominant mode with P-2 excitation. The optimal dimensions of the proposed dual-CP antenna are as follows: $W_s = 44$, $h = 1.52$, $w = 20.3$, $t = 1.9$, $g = 1.7$, $l_p = 12$, $w_p = 3$, $l_f = 12$, $s = 6$, $w_f = 1.2$ (unit: mm).

**Fig. 2:**
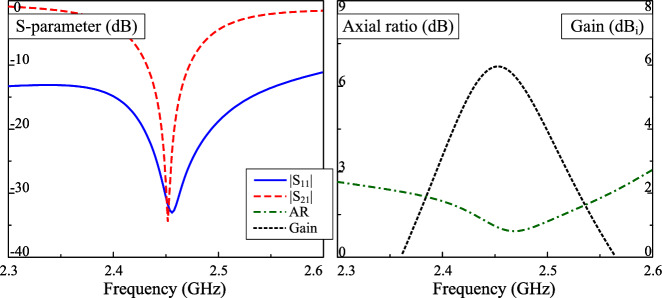
Simulated performance of the dual-CP antenna.

**Fig. 3:**
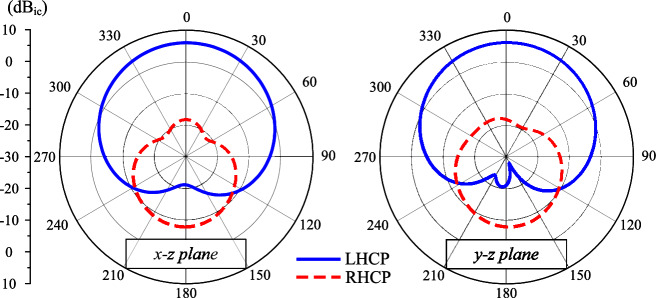
Simulated gain radiation patterns at 2.45 GHz.

The simulated S-parameter and broadside gain of the dual-CP antenna are presented in Fig. 2. It is observed that the simulated reflection coefficient ($|S_{11}|$) and transmission coefficient ($|S_{21}|$) are less than –10 dB around 2.45 GHz. Across this band, the axial ratio values are less than 3 dB, which demonstrate the CP realization of the proposed antenna. The simulated gain radiation patterns at 2.45 GHz in two principle planes are plotted in Fig. 3. The data confirm that LHCP is dominant radiation when the antenna is fed at P-1. The polarization isolation in the broadside direction ia about 25 dB, while the front-to-back ratio is about 14 dB. The back radiation can be further suppressed with larger ground plane. However, this makes the antenna size bigger, which is not the target of the proposed work.

## High-gain CP MIMO antenna

**Fig. 4:**
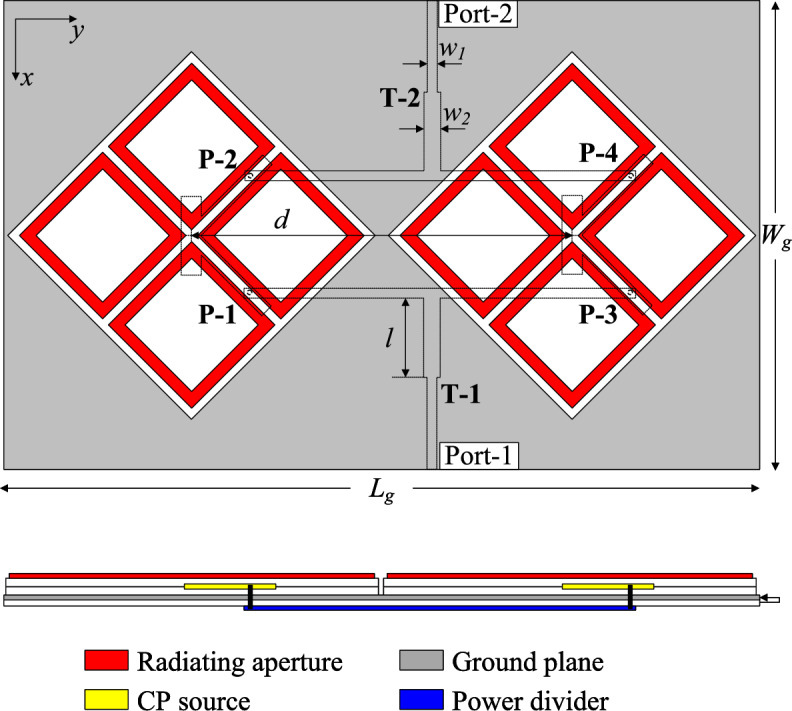
Geometry of the proposed 2-port high-gain CP MIMO antenna.

The geometry of the proposed high gain CP MIMO antenna is shown in Fig. 4. The antenna consists of two dual-CP radiators and two T-junction power dividers (T-1 and T-2). The dividers are printed on a low-cost 0.8-mm-thick FR-4 substrate with dielectric constant of 4.4. To achieve identical results for both MIMO ports, the radiators are rotated by $45^\circ$. The output ports of T-1 are connected to P-1 and P-3, while those of T-2 are linked to P-2 and P-4. It means that the 2-port MIMO antenna can radiate LHCP waves with Port-1 and RHCP waves with Port-2. When Port-1 is excited, both MIMO elements will be excited simultaneously. Accordingly, high gain radiation will be attained. The optimal dimensions of the antenna are $W_s = 44$, $h = 1.52$, $w = 20.5$, $t = 1.9$, $g = 1.7$, $l_p = 12$, $w_p = 3$, $l_f = 12$, $s = 6$, $w_f = 1.2$, $l = 14.3$, $w_1 = 1.5$, $w_2 = 2.6$, $d = 65$ (unit: mm).

**Fig. 5:**
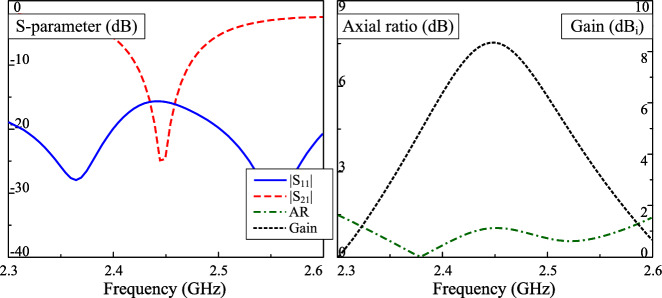
Simulated performance of the proposed high-gain CP MIMO antenna.

**Fig. 6:**
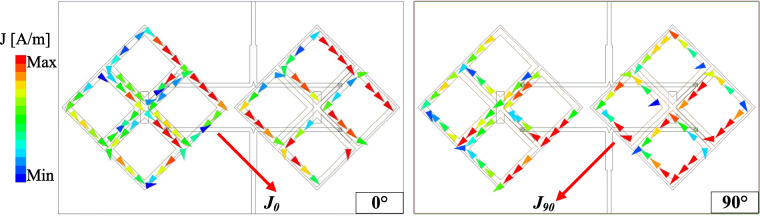
Simulated surface current distribution on the radiating apertures of the proposed antenna at 2.45 GHz.

The simulated performance of the proposed high-gain CP MIMO antenna is presented in Fig. 5. The operating bandwidth, in which the isolation is better than 10 dB, impedance matching lower than –10 dB, as well as AR is smaller than 3 dB, is from 2.43 to 2.48 GHz. The peak gain value is about 8.4 dBi at 2.45 GHz, which is about 2.4 dBi higher than that of the antenna presented in the previous section. The CP realization of the proposed can be observed in Fig. 6, which shows the simulated current distribution on the radiating apertures. As seen, when the phase changes from $0^\circ$ to $90^\circ$, the vector current rotates in the clockwise direction. Accordingly, LHCP radiation can be realized. Besides, both radiating apertures are excited simultaneously also explains for the high gain radiation. Another important radiation parameter is the radiation efficiency, which for the proposed MIMO antenna is approximately 75%.

**Fig. 7:**
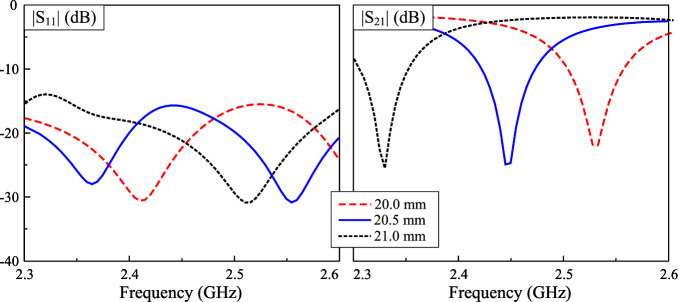
Simulated S-parameter for different values of *w*.

**Fig. 8:**
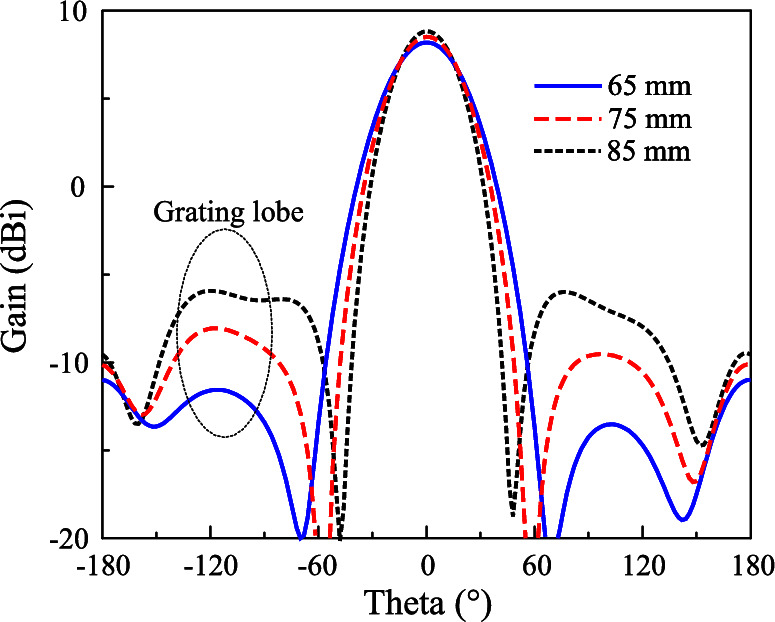
Simulated gain radiation patterns at 2.45 GHz for different values of *d*.

There are several key design parameters to optimize the proposed antenna. Firstly, the operating frequency band of the proposed design is strongly determined by the radiating aperture, *w*. As seen in Fig. 7, the operating band shifts towards higher frequency range when decreasing *w*. The reason is that smaller *w* leads to shorter path for current flowing along the ring. Next, the distance between the radiators (*d*) is considered. This parameter has minor effect on the S-parameter and AR, it only has strong effect on the gain radiation pattern of the antenna. Figure 8 shows the simulated radiation patterns at 2.45 GHz for different values of d. The broadside gain is slightly increased when the distance increases. However, this causes a higher grating lobe level. This is due to the orientation of the radiating elements, which are rotated by $45^\circ$ to ensure the identical radiation characteristics of both MIMO ports. This kind of arrangement results in large element spacing of greater than half wavelength. Here, the smallest distance of $d = 65$ mm is chosen as the optimal value for the lowest grating lobe level. Meanwhile, larger *d* results in higher gain due to the increase in array radiating aperture.

**Fig. 9:**
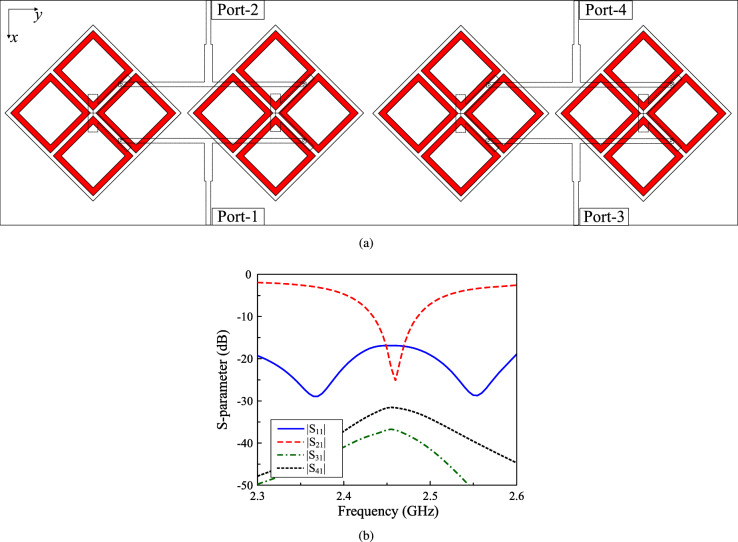
(**a**) Geometry and (**b**) simulated S-parameter of the 4-port MIMO antenna.

Next, the operation characteristics of the proposed approach when designing 4-port MIMO array are considered. Figure 9 shows the geometry and S-parameter results of the 4-port MIMO array. Obviously, good operation characteristics are also obtained around 2.45 GHz. Further simulation with multi-port MIMO of 6 and 8 ports also achieve good performance around the target frequency band. This demonstrates the effectiveness of the proposed approach for large scale MIMO array system.

## MIMO diversity performance

To evaluate the suitability of the proposed 2-port high-gain antenna for deployment in MIMO systems, its diversity performance must be thoroughly assessed. This analysis focuses on four key metrics: Envelope Correlation Coefficient (ECC), Diversity Gain (DG), Total Active Reflection Coefficient (TARC), and Channel Capacity Loss (CCL).

**Fig. 10:**
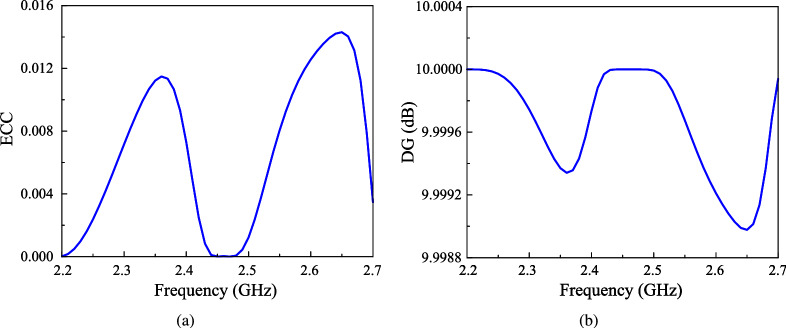
Calculated (**a**) ECC and (**b**) DG of the proposed antenna.

The Envelope Correlation Coefficient (ECC) and Diversity Gain (DG) are two critical metrics for evaluating MIMO antenna performance. ECC measures the correlation or isolation between antenna elements, with lower values ideally below 0.5 indicating reduced interference and better signal diversity. It is commonly calculated using S-parameters, as shown in equation (1). DG, on the other hand, quantifies the improvement in signal-to-noise ratio (SNR) achieved through diversity techniques, which is essential for reliable communication in challenging environments like multipath fading. Since DG depends on ECC, it is calculated by using a relationship shown in equation (2), with values close to 10 dB indicating minimal correlation and optimal performance. These metrics ensure robust MIMO operation, as concluded by the simulation results in Fig. 10a and b, where low ECC and high DG values highlight the antenna’s ability to deliver high-quality signal transmission with negligible disruptions.

1 $$\begin{aligned} ECC= & \frac{{{{\left| {S_{11}^*{S_{12}} + S_{21}^*{S_{22}}} \right| }^2}}}{{\left( {1 - {{\left| {{S_{11}}} \right| }^2} - {{\left| {{S_{21}}} \right| }^2}} \right) \left( {1 - {{\left| {{S_{22}}} \right| }^2} - {{\left| {{S_{12}}} \right| }^2}} \right) }} \end{aligned}$$

2 $$\begin{aligned} DG= & 10\sqrt{1 - {{(ECC)}^2}} \end{aligned}$$

**Fig. 11:**
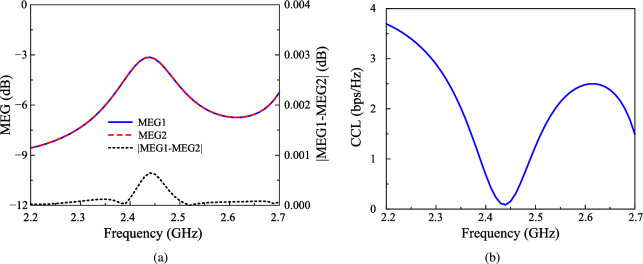
Calculated (**a**) MEG and (**b**) CCL of the proposed antenna.

Beyond ECC and DG, Mean Effective Gain (MEG) is another vital metric for evaluating how well an antenna performs in real-world environments, particularly in MIMO systems. MEG measures the average power received by an antenna compared to an ideal isotropic radiator, factoring in the antenna’s radiation pattern and the angular distribution of incoming signals. This makes MEG especially important for understanding how effectively an antenna can capture signals in complex, multipath environments, which directly impacts data rates and communication reliability. A popular method to determine MEG is using S-parameters as expressed in equation (3), which describe power coupling between antenna ports. For optimal performance, the MEG of each port should ideally range between -3 dB and -10 dB to ensure efficient power reception, and the MEG difference between any two ports should not exceed 3 dB to maintain balanced performance across the antenna array. An observation to Fig. 11a claims that the calculated results for the proposed antenna satisfy these requirements. Alongside MEG, the reduction in channel capacity due to mutual coupling and correlation between antenna elements is evaluated via Channel Capacity Loss, where CCL is derived from the determinant of the correlation matrix and is expressed in bits per second per Hertz (bps/Hz), as shown in equation (4). A lower CCL value, typically below 0.4 bps/Hz, indicates better data transmission efficiency, as demonstrated by the proposed MIMO antenna in Fig. 11b, which maintains CCL values well below this threshold across the target frequency band.

3 $$\begin{aligned} & \mathrm{{ME}}{\mathrm{{G}}_i} = 0.5\left[ {1 - \sum \limits _{j = 1}^N {{{\left| {{S_{ij}}} \right| }^2}} } \right] \end{aligned}$$

4 $$\begin{aligned} & \begin{array}{l} CCL = - \log _2^{\left| {{\psi ^R}} \right| }\\ Where\mathrm{ }{\psi ^\mathrm{{R}}} = \left[ {{\rho _{\mathrm{{ij}}}}} \right] ,(\mathrm{{i}},\mathrm{{j}}) \in (1,2)\\ and\mathrm{ }{\rho _{11}} = \left( {1 - {{\left| {{S_{11}}} \right| }^2} - {{\left| {{S_{12}}} \right| }^2}} \right) \\ \mathrm{ }{\rho _{22}} = \left( {1 - {{\left| {{S_{21}}} \right| }^2} - {{\left| {{S_{22}}} \right| }^2}} \right) \\ \mathrm{ }{\rho _{12}} = \left( {S_{11}^*{S_{12}} - S_{21}^*{S_{22}}} \right) \\ \mathrm{ }{\rho _{21}} = \left( {S_{22}^*{S_{21}} + S_{12}^*{S_{11}}} \right) \end{array} \end{aligned}$$

**Fig. 12:**
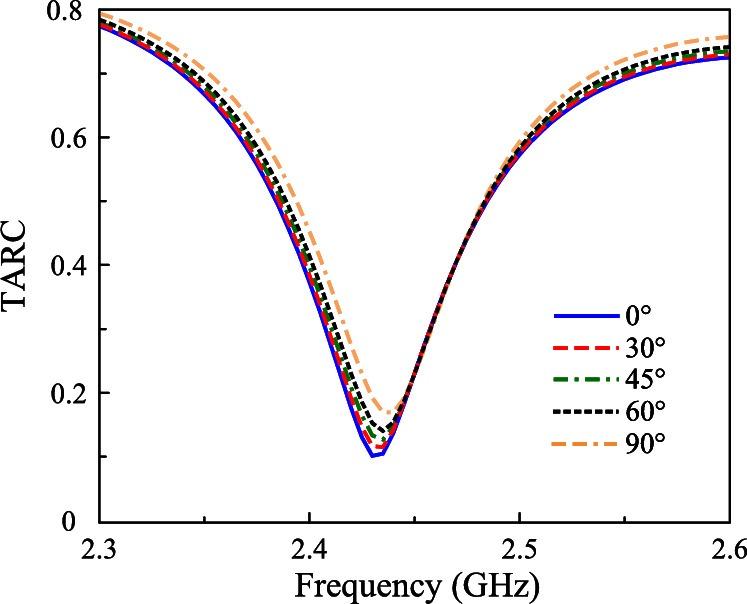
Calculated TARC of the proposed antenna with different incident angles.

Finally, the Total Active Reflection Coefficient (TARC) is considered. This kind of parameter shows the efficiency of the MIMO system when all ports are excited simultaneously. The calculated formula for TARC is shown in equation (5). TARC should be as close to 0 as possible, ensuring minimal power reflection and maximum transmission efficiency of the MIMO system. As shown in Fig. 12, which shows the TARC for different incident angles, the TARC values are quite similar for all cases and they are all around 0.15.

5 $$\begin{aligned} \text {TARC} = \sqrt{\frac{|S_{11} + S_{12}e^{j\theta }|^2 + |S_{21} + S_{22}e^{j\theta }|^2}{2}} \end{aligned}$$

**Fig. 13:**
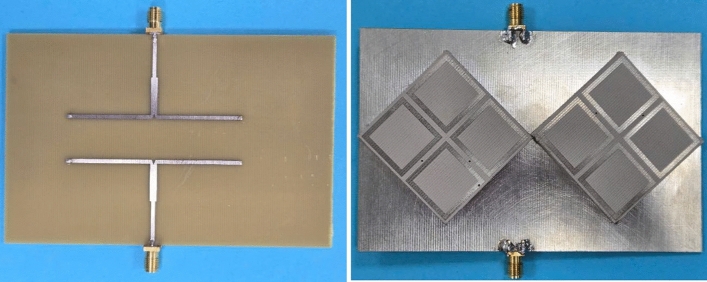
Photographs of the fabricated antenna. The left one is the bottom view (T-divider), the right one is the top view (radiating elements and ground).

**Fig. 14:**
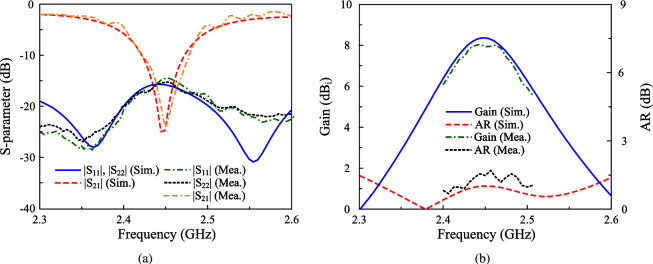
Simulated and measured performance of the proposed high-gain CP MIMO antenna. (**a**) S-parameter, (**b**) Gain and AR with Port-1 excitation.

**Fig. 15:**
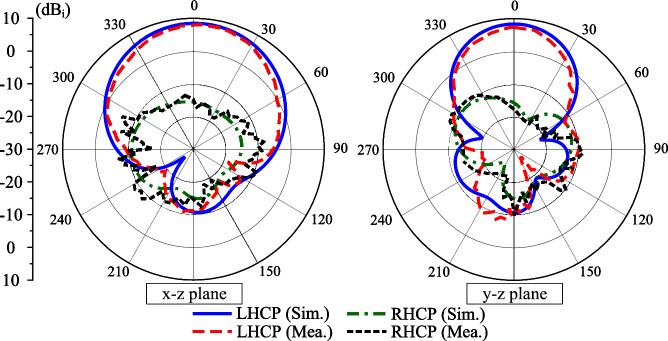
Simulated and measured gain radiation patterns at 2.45 GHz.

**Table 1:** Performance comparison among 2-port high-gain CP MIMO antennas.

Ref.	Overall size ($\lambda$)	Radiator	No. of radiating elements	High gain approach	BW (%)	Isolation (dB)	Peak gain (dBi)
^16^	6.00 $\times$ 3.00 $\times$ 0.03	Single-pol.	8	T-divider	0.8	42	14.6
^17^	2.80 $\times$ 1.40 $\times$ 0.09	Single-pol.	8	T-divider	17.7	42	11.5
^18^	1.87 $\times$ 0.54 $\times$ 0.03	Single-pol.	4	T-divider	1	40	5.4
^19^	6.00 $\times$ 2.30 $\times$ 0.08	Single-pol.	4	T-divider	8.7	62	9.5
^20^	1.82 $\times$ 1.82 $\times$ 0.25	Single-pol	2	FSS layer	2	18	9.8
^21^	2.06 $\times$ 2.06 $\times$ 0.10	Single-pol	2	FSS layer	3.5	17	9.1
Prop.	1.06 $\times$ 0.65 $\times$ 0.03	Dual-pol.	2	T-divider	1.6	30	8

## Measurement results

The proposed concept is validated by measuring the fabricated antenna prototype, as depicted in Fig. 13. The simulated and measured S-parameter and AR shown in Fig. 14 indicate that the operating bandwidth of the proposed antenna is from 2.43 to 2.485 GHz, in which the reflection and transmission coefficients are well below -10 dB and the AR is lower than 3 dB. Across this band, the broadside gain is about 8.0 dBi. Furthermore, The measured radiation patterns at 2.45 GHz with Port-1 excitation as illustrated in Fig. 15 show good broadside beam. The polarization isolation in the forward direction is better than 22 dB and the front-to-back ration is about 18 dB.

The performance comparison among the 2-port high-gain CP MIMO antennas using microstrip patch structure is summarized and given in Table 1. In this Table, the “Number of radiating elements” column indicates the total elements employed for the 2-port MIMO designs. It is obvious that the proposed design has the smallest size with the lowest required number of radiating elements. It comes from the utilization of dual-polarization radiator. The designs in^16,17^ can achieve higher gain due to the use of 4 radiators for one MIMO port. These structures suffer from an extremely large antenna dimensions, which are not suitable for compact devices. Meanwhile, the MIMO arrays in^18,19^ employ 2 radiators for one MIMO port, which is similar to the proposed work. However, the number of radiating elements for the proposed work is just half of those in^18,19^, while achieving comparable gain. Compared to the gain enhancement approach of using FSS layers^20,21^, the proposed design has smaller size and much lower profile.

## Conclusion

This paper presented the approach to design compact high gain CP MIMO antenna. The proposed approach is a combination of two dual-CP radiators and two T-junction power dividers. The measured on fabricated antenna has operating bandwidth from 2.43 to 2.485 GHz isolation of better than 10 dB and peak gain value of 8.0 dBi. The antenna also performs good MIMO diversity performance with respect to ECC, DG, MEG, CCL, and TARC. Unlike the conventional approach of using T-divider and single-polarized radiator, which causes large overall size due to the use of multiple radiating elements, the proposed approach can achieve high gain with smaller number of radiating elements, which results in more compact overall dimensions. The proposed antenna can be a potential candidate for WLAN applications operating at 2.45 GHz.

## Data Availability

Data is provided within the manuscript.
